# Embed circular economy thinking into building retrofit

**DOI:** 10.1038/s44172-022-00027-2

**Published:** 2022-11-03

**Authors:** Danielle Densley Tingley

**Affiliations:** Department of Civil and Structural Engineering, Sir Frederick Mapping Building, Mappin Street, The University of Sheffield, Sheffield, S1 3JD UK

**Keywords:** Energy efficiency, Climate-change mitigation, Environmental impact

## Abstract

Building retrofit is essential to deliver decarbonisation. But its implementation could leave a legacy of waste if end of life is not considered now. Danielle Densley Tingley considers the challenges and implications of embedding circularity into building retrofit.

## Teaser

Building retrofit is essential to deliver decarbonisation. But its implementation could leave a legacy of waste if end of life is not considered now. Here I consider the challenges and implications of embedding circularity into building retrofit.

## Introduction

Buildings retrofits improve energy efficiency and are an essential component of global decarbonisation plans. However, whilst many retrofit techniques will reduce energy demand in the near future, their longevity is rarely considered. This risks a legacy of waste with high recurring embodied carbon, and buildings that cannot be easily further upgraded in decades to come. To avoid this, engineers, architects, designers and builders must start now to embed a circular economic (CE) approach into building retrofit.

## Typical retrofits

Retrofits usually tackle two main components: the building fabric and building services. I will focus on domestic building fabric measures. These seek to improve the thermal efficiency of walls, windows and doors through installation of insulation, double or triple glazing, and insulated doors. In temperate climates, such as the UK, building engineers and designers typically focus on reducing heat loss from the building to reduce energy demand. As temperatures rise however, they should also be aware of the post-retrofit risk of overheating in summer months and should ensure sufficient ventilation is provided.

The thermal performance of building fabric retrofits typically degrades over time. For example, organic, closed cell insulation materials such as expanded polystyrene, extruded polystyrene, polyurethane, and phenolic foam, leach their foaming gases over time, reducing their thermal performance^1^. Similarly double glazing will not indefinitely retain its thermal performance. Over time glazing seals fail, which allow fresh air into the air gap between the glazing panes. This reduces thermal efficiency and can result in condensation between the glazing panes.

In both cases the degradation in performance could lead to the need for replacement of insulation or glazing to optimise buildings’ thermal efficiency. The degradation in performance of glazing means that windows are typically replaced after 20–30 years. While the component materials (e.g. plastic frame and window glass) can be recycled, this requires energy and supply chains to ‘take-back’ the materials. Glazing replacement is more frequent as the condensation highlights the degradation. But in a future net zero world, we are likely to also seek to replace insulation more often to improve building energy efficiency.

## The value of applying circular economy principles

CE aims to keep materials at the highest value possible. In the case of a building this means retention and retrofit (rather than demolition) for as long as possible. However, we also need to consider how to keep the components and materials that are used in retrofit at the highest value possible. This means rather than recycling the materials from a window, priority should be placed on remanufacturing the window in-situ. This retention of materials results in lower greenhouse gas emissions from the production and transportation of materials (initial embodied carbon) than replacement. Similarly for insulation, new wall designs and products should allow for easy replacement of insulation. At the same time, there needs to be an insulation system that avoids insulation contamination so it can be recovered and recycled when its performance is degrading.

A shift to a CE retrofit approach will likely have implications for the stakeholders involved. Ideally builders would retain information via a retrofit passport, the technical feasibility of which has already been explored in the EU^2^. A retrofit passport could be passed between homeowners on property sale and would give homeowners greater insight into existing retrofit measures, including how to efficiently upgrade them when required. A circular retrofit would make it easier for homeowners to carry out upgrades, for example through on-site remanufacturing, and any resulting waste streams would not be contaminated and thus could be recycled through take-back schemes. Supply chains must also be better established to make this easier for consumers. It’s clear that a shift to a circular retrofit model would also require large scale upskilling of a very fragmented workforce.

Whilst a circular retrofit approach does present potential challenges, the potential carbon benefits are significant. Li et al.^3^ modelled the domestic retrofit measures that are required for England to stay within carbon budgets. They found that for all eligible properties a package of retrofit measures needed to be installed in 2021, including external or internal or cavity wall insulation, double glazing and heat pumps. Different material choices and different rates of grid decarbonisation give opportunities to stay within carbon budgets with a slower, more practical retrofit installation rate. But the urgent need to retrofit cannot be understated. Circular retrofits need to be implemented rapidly and at scale. If this opportunity is missed, the future waste burden will likely be considerable. To put this into context, using Li et al.^3^ as a basis, a wall area of ~3 billion m^2^ requires insulating, covering cavity wall, external and internal insulation. If an average thickness of 100 mm is assumed, this equates to a volume of 300 million m^3^ of insulation that is required to insulate the walls of England’s housing stock. This is a tremendous quantity of material that will also need end of life processing as landfill or via incineration if circular retrofit practices are not adopted.

## How do we embed CE thinking into retrofit practices?

There are a number of strategies that have been set out to accelerate retrofit across the UK for example, the UK Green Building Council’s Retrofit Playbook^4^. It would therefore be logical to integrate CE thinking into the pre-existing approaches suggested to accelerate retrofit. These plans typically encompass the following five areas: (1) finance and incentives, (2) technology, (3) skills and supply chain capacity, (4) policy, and (5) engagement with consumers. Implications of a CE approach on each of these areas are discussed in the sections below.

### Finance and incentives

How retrofits are financed is a critical question with which successive governments and retrofit stimulus packages have struggled. Putting a CE lens on the question introduces more financial options. For example, products as a service is a CE concept, in which rather than buying a product outright, you regularly pay for the service the product provides. In retrofit terms, this could mean paying a yearly fee for a warm home, or heat. Theoretically a supplier would install fabric efficiency and energy supply measures to deliver a warm home. The occupier would pay an agreed yearly fee for this warm home. The supplier is then incentivised to ensure on-going fabric efficiency and operational efficiency of the installations continues, as otherwise they would be responsible for increasing energy use costs. This means the supplier is more likely to replace elements as required to maintain the performance. They are then in a position to remanufacture these elements for reuse, or recycle materials back into their supply chain to reduce costs. This is a simplification of an incredibly complex scenario, the economics and business case of these requires research, particularly given the 20–30 year lifespans of retrofit products, and increasing fuel prices. Furthermore, implementation of performance-based models would be challenging as they hold manufacturers and installers to a performance level—which is currently rarely the case. Post-retrofit performance is seldom tested. The EnerPHiT standard does require an airtightness test post-retrofit^5^, but only a small sample of retrofits seek this certification. Nevertheless, this illustrates how CE thinking could also offer new financial opportunities to stimulate retrofit.

### Technology

**Fig. 1:**
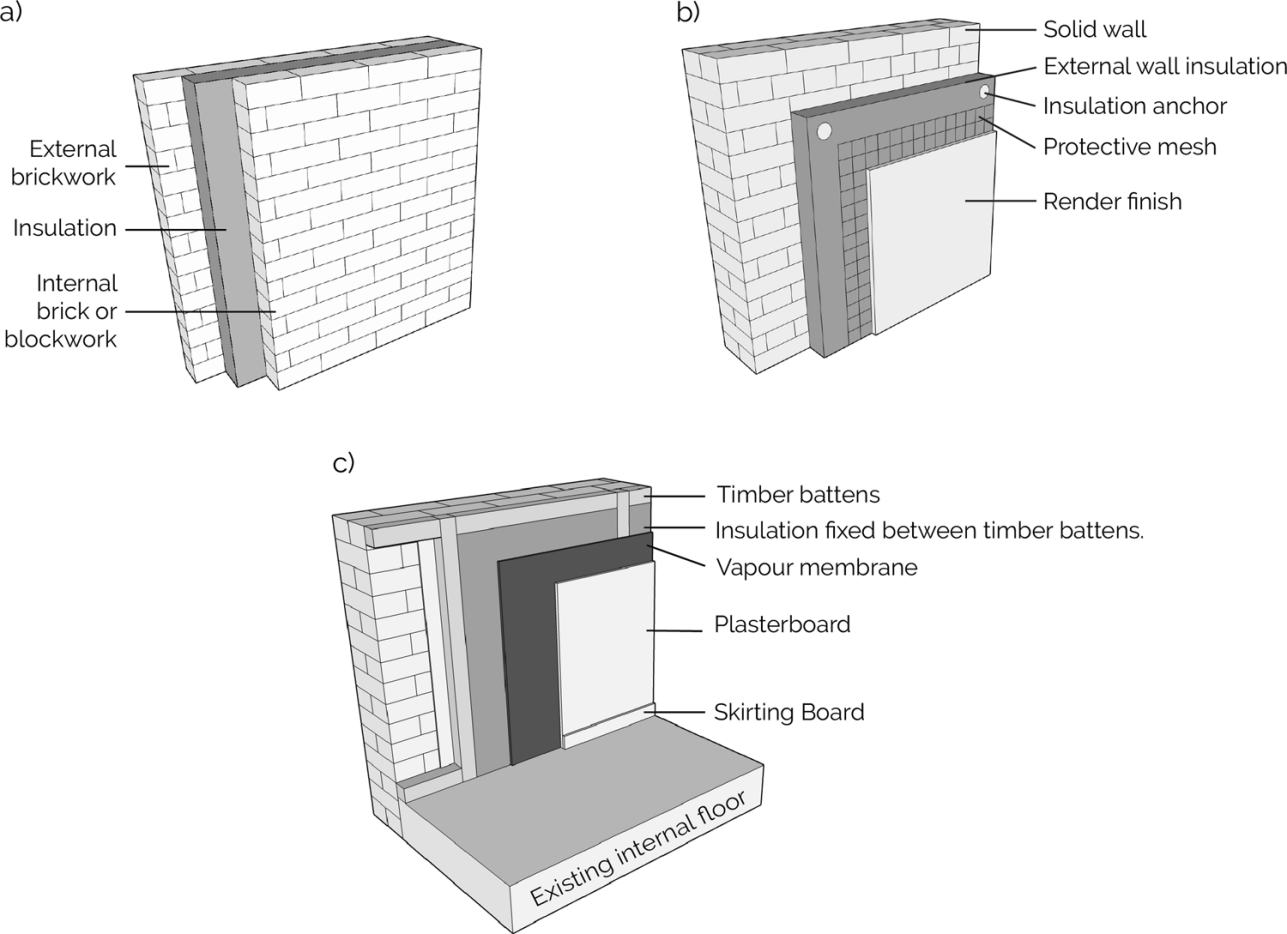
Examples of wall insulation details. **a** An example of wall insulation details, with insulation fitted between two layers of masonry construction, brick and brick, or brick and concrete block. **b** An example of external wall insulation: insulations typically glued and mechanically fixed to a concrete wall. **c** An example of internal wall insulation. Insulation can be directly glued and/or mechanically fixed to the wall, or fixed between, or onto timber battens which are attached to the wall.

Retrofit products and systems need to be easily upgraded, remanufactured and reused, or at least the components recycled. Fig. 1 shows three typical wall insulation methods in the UK. There are different challenges for implementing CE solutions across each of these methods. When retrofitting cavity wall insulation (Fig. 1a), holes are typically drilled into the brick to access the cavity, insulation is then blown into this existing cavity. Upgrades would typically involve blowing more insulation into the cavity. This nature of construction makes it challenging to retrofit a CE solution—e.g. where the existing insulation material can be removed and recycled.

External wall insulation (Fig. 1b) presents different CE challenges. A CE solution requires reversible, mechanical connections so insulation can be easily removed and replaced. The mesh and render finish will be very difficult to remove from the insulation, contaminating it and likely prohibiting recycling at end of life. A CE solution is more likely to be a mechanically attached, layered panel, that can be removed and reused, or components mechanically separated and the insulation recycled and replaced so remaining panel elements can be reused.

A CE approach to internal wall insulation (Fig. 1c) again must move away from adhesive connections and finishes to mechanical ones. The typical plaster skim finish to plasterboard makes it challenging to remove or replace any layers without damage. A CE solution could have a panelised finish, or tape and joining of plasterboard to avoid the plaster skim. Screwing timber battens into the wall and close fitting of insulation between these would provide a reversible solution, and the vapour membrane will likely need to be taped and pinned, rather than stapled.

Developing CE retrofit approaches is an urgent interdisciplinary challenge, one that requires collaboration from academia and industry to develop solutions, pilots and demonstrator studies to test and showcase new technologies. This approach is being taken in the Dutch REHAB research project that is developing ‘Circular Skin’ systems^6^. Once solutions are shown to be viable, they must be deployed at scale to market.

### Supply chain skills and capacity

A lack of specialist retrofit skills, and capacity within the construction sector are also barriers to implementation^4^. If there is to be a skills development programme for retrofit, then this should include an understanding of CE principles, and their application in this context. The resulting workforce would then be equipped to conduct circular, energy-efficient retrofits, and understand the importance of recovering materials for reuse/remanufacturing/recycling. From a design perspective many universities are increasing CE teaching within their architecture and engineering programmes, which should increase the awareness of those moving into these professions. In addition, the Circular Economy Club lists a number of MSc/MBA programmes^7^ which place the circular economy at their heart. Furthermore, developing the product supply chain for circular retrofits, alongside the technology development, will be essential for at scale deployment. Scale is critical. There are nearly 25 million homes in England alone, the majority of which will require some level of retrofit to deliver a net zero UK. There needs to be sufficient product supply to enable circular retrofits. The material demand implications of these also need to be understood, alongside quantification of the total embodied emissions, as already conducted by Li et al. for ‘typical’ retrofits.

End of life of both ‘typical’ and ‘circular’ retrofits must also be considered. There is a question of who takes responsibility for end of life remanufacturing, reuse, recycling or disposal. Should the responsibility lie with the manufacturer? End of life regulations, and producer responsibility exists for vehicles^8^, so should they for buildings and retrofits? A shift to producer responsibility for retrofit products implies a new role in the construction supply chain: an end of life specialist who can upgrade, or deconstruct and recover components from retrofits. It might be that installers also can evolve to conduct this role, but it would still require skills development. Producers would need to establish take-back schemes to facilitate remanufacturing and reuse, or recycling of different products. A further challenge is how the varying lifespans and technical specification of different retrofit products can be taken into account in take-back schemes.

### Policy

UK policy to encourage and support retrofit is severely lacking given net zero ambitions by 2050. This means that there is an opportunity to embed CE thinking into any upcoming retrofit policies; this could be at local or national levels. Policies might include financial incentives, such as discounts for CE retrofits, stamp duty discounts/rebates if CE retrofit measures are implemented before selling a home, or within a set number of years of buying a new home, and VAT discounts for CE retrofit products.

Consistency and potentially mandating minimum product lifetime guarantee periods would also be beneficial. For example, all fabric efficiency retrofit products should maintain their thermal efficiency for a minimum of 30 years, as already offered by ‘whole house retrofit’ firms like Energiesprong^9^. Debate can of course be had over the particular lifetime chosen. But a minimum threshold would assist consumers in installing products that will last. How the measure can be replaced or upgraded after the specified lifespan likely also needs legislation. This could be in the form of producer responsibility, as discussed in the ‘Supply Chain Skills and Capacity’ section.

### Engagement with consumers

Engaging with consumers on a CE approach will be similar to engaging with consumers on retrofit more generally. The UKGBC Retrofit Playbook outlines typical means of doing this for local authorities. The key difference with a circular retrofit is most likely that it can be easily upgraded, which will be more appealing to some owners than others given upgrades will likely be 30 years into the future. Given that this benefit will arise in the future, incentivising a CE retrofit for many consumers could be a challenge. This is often found to be the case with CE more generally. This is where policy or financial incentives, as discussed in the sections above, could play a role.

## Summary and outlook

So far I have focused on the UK context. But much of what I have discussed will be applicable to inefficient building stocks internationally, particularly those that have a larger demand for heating than cooling, for example, many countries in Northern Europe. In a Dutch context, van Stijn and Gruis^10^ discussed the concept of modular, mass-customisable and cyclable retrofit products that could replace different layers of Dutch buildings, exploring the concept further for circular kitchens. The same group are also exploring designs and building prototypes for circular skins, based on extendible modules so renovations can happen in stages^6^.

The challenges I have discussed are also applicable to facade design of new buildings. Circular thinking must be embedded into these new designs as well, facilitating future upgrades to maintain operational efficiency, whilst maximising resource efficiency. Hartwell et al.^11^ explored the challenges and opportunities of new circular facade design through stakeholder surveys and interviews. The recommendations highlighted by Hartwell et al. draw some parallels with this commentary, in particular training for the supply chain, and supply chain infrastructure to facilitate reuse.

There is much work to be done. The critical next steps I have outlined will require huge efforts from engineers, supply chain experts, educational institutions, policymakers, building and construction firms and product manufacturers. However, now is a pivotal moment, given the widespread, acknowledged need for retrofit to directly tackle both the climate emergency and control heating costs far into the future.
